# Long non-coding RNA Lucat1 is a poor prognostic factor and demonstrates malignant biological behavior in clear cell renal cell carcinoma

**DOI:** 10.18632/oncotarget.21185

**Published:** 2017-09-23

**Authors:** Haibing Xiao, Lin Bao, Wen Xiao, Hailong Ruan, Zhengshuai Song, Yan Qu, Ke Chen, Xiaoping Zhang, Hongmei Yang

**Affiliations:** ^1^ Department of Pathogenic Biology, School of Basic Medicine, Tongji Medical College, Huazhong University of Science and Technology, Wuhan 430030, China; ^2^ Department of Urology, Union Hospital, Tongji Medical College, Huazhong University of Science and Technology, Wuhan 430022, China

**Keywords:** Lucat1, EZH2, p57

## Abstract

**Background:**

Many long intergenic noncoding RNAs (lincRNAs) are encoded in the human genome. However, their biological functions, molecular mechanisms and prognostic values associated with clear cell renal cell carcinoma (ccRCC) have yet to be elucidated.

**Methods:**

We screened the lncRNAs’ profile in ccRCC from The Cancer Genome Atlas (TCGA) database, and selected Lucat1 for further study. MTS, colony formation assay and transwell assay were performed to examine the effect of Lucat1 on proliferation and metastasis of ccRCC. The Chip and Rip assay was performed to verify that Lucat1 can bind to polycomb PRC2 complex and suppress p57 expression.

**Results:**

In this study, we found that lncRNA Lucat1 expression was significantly up regulated in tumor tissues compared to matched adjacent non-tumor tissues. The Lucat1 expression level was also associated with grade, the clinical pathological stage and the survival time. Functional assays showed that Lucat1 can promote renal cancer cell proliferation *in vitro* and *in vivo*. Further analysis showed that Lucat1 can bind to polycomb PRC2 complex and suppress p57 expression.

**Conclusions:**

Taken together, our results suggest that Lucat1, as a regulator of proliferation, may serve as a candidate prognostic biomarker and target for novel therapies in human ccRCC.

## INTRODUCTION

The rate of renal cell carcinoma (RCC) has been rising throughout the world. Renal cell carcinoma (RCC) accounts for nearly 5% of adult malignancies with about 63,920 new cases and 13,860 deaths estimated in 2014 in the United States [1]. In China, about 66,800 new cases and 23,400 deaths were estimated for 2014 [2]. Clear cell renal cell carcinoma (ccRCC) is the most common subtype and represents approximately 75% of all renal tumors. Metastasis is common in ccRCC, and approximately one-third of ccRCC patients have metastasis at the time of diagnosis despite the wide use of ultrasound and computed tomography [3]. In addition, renal cancer patients respond poorly to radiation treatment and conventional chemotherapy [4]. Hence, a better understanding of the mechanisms involved in the pathogenesis of ccRCC and more effective therapeutic approaches are crucial [5].

Long non-coding RNA (lncRNA) is a heterogeneous class of transcription RNA with a minimum length of 200 bases and limited protein-coding potential [6, 7]. LncRNAs are involved in multilevel regulation of gene expression, including transcriptional regulation by recruiting chromatin-modifying complexes [8, 9] and post-transcriptional regulation by interacting with miRNAs, mRNAs, or proteins [10, 11].

Recently, numerous lncRNAs have been identified to have a direct role in recruiting PRC2. PRC2, a methyltransferase that is composed of enhancer of zeste homolog 2 (EZH2), suppressor of zeste 12 (SUZ12) and embryonic ectoderm development (EED), can catalyze the di- and trimethylation of lysine residue 27 of histone 3 (H3K27me3), thus modulating gene expression. Ahmad M. Khalil *etal* have found that nearly 20% of lincRNAs expressed in various cell types are bound by PRC2 [12]. These lncRNAs epigenetically regulate gene expression through binding to PRC2 in various biological processes, especially during cancer, such as HOTAIR, TUG1, MALAT1, PINT etc. [13–20] .

Lucat1, also named SCAL1, is induced by cigarette smoke and elevated in lung cancer cell lines [21] and may contribute to cisplatin resistance in high grade serous ovarian cancer [22]. An abstract implies that Lucat1 may decrease the expression of p21 and p57 in human non-small cell lung cancer [23]. However, the biological functions of Lucat1 in the control of ccRCC tumorigenesis remain largely unknown.

In this study, the TCGA database was used to search for lncRNA gene expression profiles in ccRCC. We identified Lucat1 as a new candidate lncRNA that promotes the development of ccRCC. Our data indicated that Lucat1 has higher expression in renal cancer cell lines and renal cancer tissues. We also found that Lucat1 is capable of facilitating cell growth, migration and invasion through epigenetically suppressing p57 in renal cancer cell lines.

## RESULTS

### Lucat1 is upregulated in ccRCC tissues and cell lines and indicates a poor prognosis

**Figure 1:**
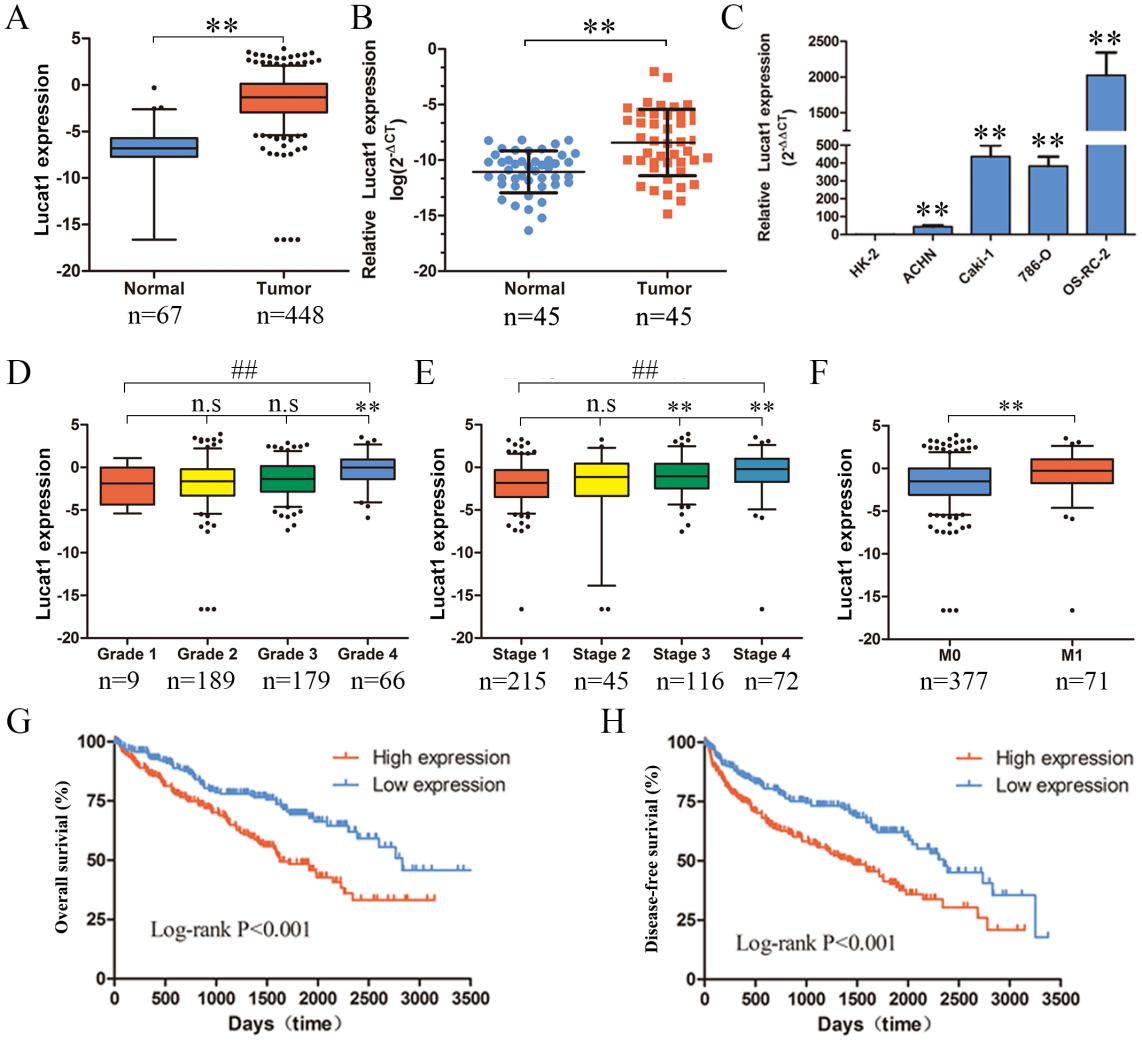
Expression of Lucat1 in ccRCC cell lines, tissues and its clinical parameters **(A)** Lucat1 was found to be highly over-expressed in ccRCC tissues compared with normal tissues in the TCGA RNA-seq data (P < 0.001). **(B)** Relative expression of Lucat1 in 45 pairs of ccRCC tumor tissues and their corresponding adjacent non-cancerous tissues. **(C)** Real-time PCR analysis of Lucat1 expression in immortalized human renal tubule epithelial cell line HK-2 and indicated renal carcinoma cell lines. **(D, E, F)** High Lucat1 expression was signifcantly correlated with the TNM grade, stage and metastasis. **(G, H)** High Lucat1 expression contributed to a significant poorer oval-all survival and disease-free survival at the TCGA database (n=480).^*^, t-test p<0.05; ^**^, t-test p<0.001; ^##^, ANOVA, p<0.001.

The TCGA database was used to search for differentially expressed lncRNAs between ccRCC tissues and normal tissues. Based on ccRCC RNA-seq data, Lucat1 was found overexpressed in ccRCC tissues compared with normal (Figure 1A). To further support this conclusion, we examined the expression of Lucat1 expression in 45 renal cancer tissues and their corresponding noncancerous tissues from Union Hosptial and obtained the same result (Figure 1B). In comparison, we also found Lucat1 was ubiquitously expressed at higher levels in a panel of 5 human clear cell renal cell carcinoma lines than immortalized human proximal renal tubule epithelial cell line HK-2 (Figure 1C). Moreover, to assess the clinical significance of Lucat1, we evaluated the correlation between its level and clinic-pathological parameters. Results revealed that Lucat1 levels were remarkably correlated with grade, TNM stage and metastasis in ccRCC (Figure 1D, 1E and 1F) (Table 1). Nevertheless, Lucat1 levels were not associated with other clinical characteristics, including gender (p = 0.115), age (p = 0.108) and recurrence (p = 0.225). Additionally, multivariate cox regression analysis revealed that high Lucat1 expression, age, TNM stage, grade, metastasis are independent predictors of OS in ccRCC patients (Table 2). Kaplan-Meier analysis indicated that high Lucat1 expression was related to a poorer overall survival (log-rank test, P <0.001, Figure 1G) and disease free survival (log-rank test, P <0.001, Figure 1H). Taken together, these results confirmed that high Lucat1 expression was related to poor prognosis, and upregulated expression of Lucat1 might be crucial in ccRCC tumorigenesis and progression.

**Table 1:** The characteristic of Lucat1 in clear cell renal cell carcinoma

Characteristic		Total (n = 448)	lncRNA-Lucat1		p Value
			Low (224)	High (224)	
Gender	Male	287	135	152	
	Female	161	89	72	0.115
Age	<=60	218	118	100	
	>60	230	106	124	0.108
T	T1&T2	276	156	120	
	T3&T4	172	68	104	0.001
N	N0	218	108	110	
	N1	16	3	13	0.017^a,c^
	Nx	214	113	101	0.031
M	M0	377	199	178	
	M1	71	25	46	0.009
Stage	1,2	260	148	112	
	3,4	188	76	112	0.001
Grade	1,2	198	111	87	
	3,4	245	109	136	0.015^b^
	X	5	4	1	0.015^c^
Recurrence	No	349	180	169	
	Yes	99	44	55	0.225

a: N0, N1 and Lucat1 expression, b: Grade1,2 Grade3,4 and Lucat1 expression, c: Fisher Two-tailed Fisher’s exact test.

**Table 2:** Univariate and multivariate analyses of clinicopathological factors for overall survival

Risk factors	Univariate analysis			Multivariate analysis		
	HR	p-value	95% CI	HR	p-value	95% CI
Lucat1 expression	1.155	0.000^**^	1.082-1.233	1.124	0.002^**^	1.043-1.211
Age	1.695	0.002^**^	1.220-2.354	1.488	0.018^*^	1.070-2.070
Grade	2.421	0.000^**^	1.692-3.463	1.489	0.040^*^	1.017-2.179
Stage	4.308	0.000^**^	3.046-6.095	2.466	0.000^**^	1.624-3.744
M	4.765	0.000^**^	3.424-6.632	2.299	0.000^**^	1.564-3.380
T	3.597	0.000^**^	2.588-5.001			
N	2.821	0.002^**^	1.484-5.365			
Recurrence	2.407	0.000^**^	1.745-3.319			
Gender	1.022	0.896	0.737-1.418			

### Knockdown of Lucat1 inhibited cell proliferation and metastasis *in vitro*

**Figure 2:**
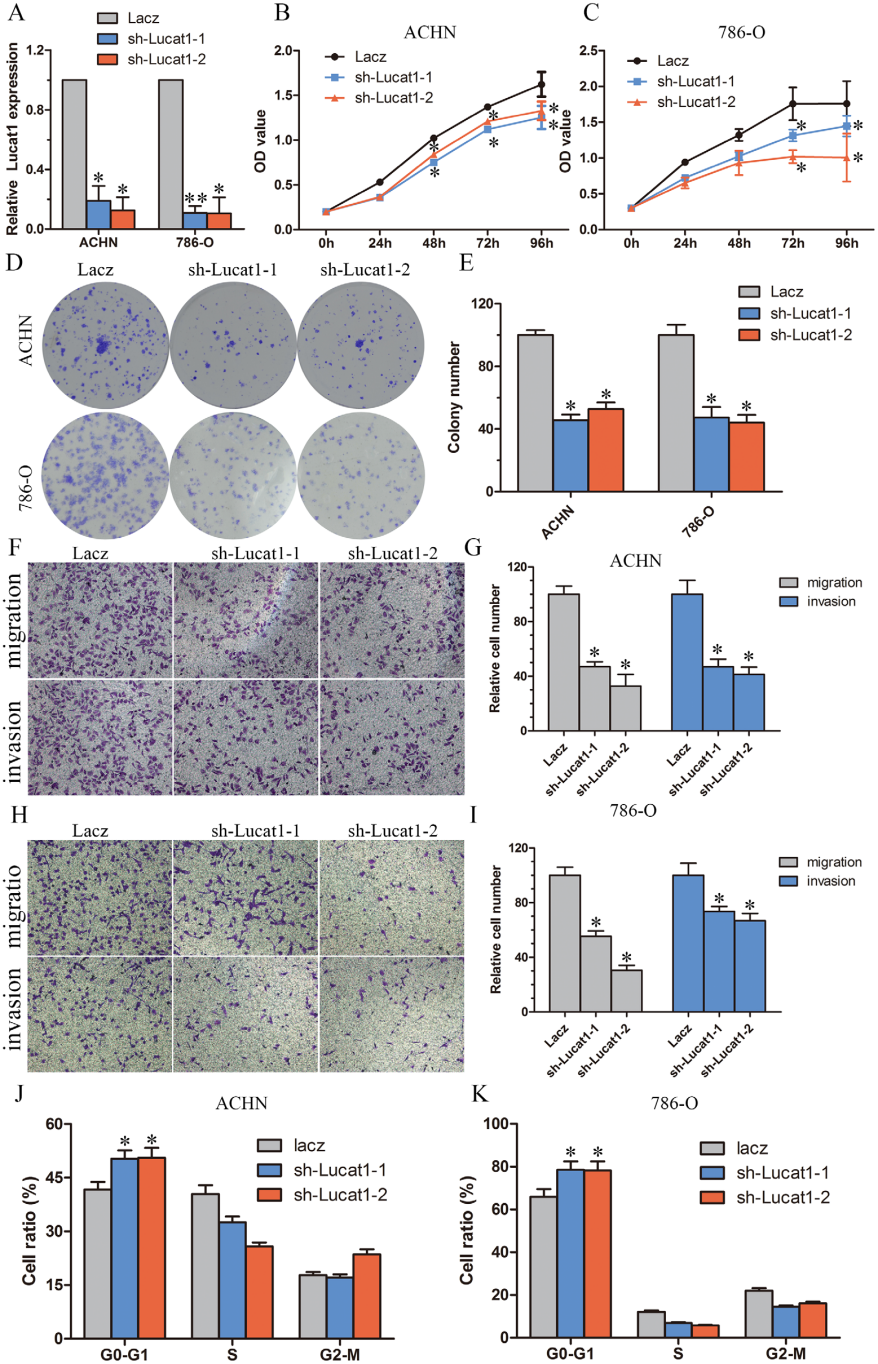
Knockdown of Lucat1 inhibited cell proliferation and metastasis *in vitro* **(A)** The effciency of Lucat1 silencing in short hairpin RNA-stably transduced renal cancer cell lines ACHN and 786-O. Relative gene expression was determined using the comparative delta-delta CT method (2-ΔΔCt). **(B, C)** MTS assays revealed cell growth curves of indicated cells. **(D, E)** Representative micrographs of crystal violet-stained cell colonies analyzed by clongenic formation and its relative count. **(F, G, H, I)** Migration and invasion assay for renal cancer cells. Representative photographs were taken at × 200 magnifcation; number of migrated cells was quantified in ten random images from each treatment group. **(J, K)** Flow cytometric determination of proportion of indicated cells in distinct cell cycle phases.

To explore the role of Lucat1 in renal cancer cells, we stably inhibited Lucat1 in two ccRCC cell lines ACHN and 786-O with lenti-viruses carrying shRNA for Lucat1 and a control nonspecific shRNA (LacZ) (Figure 2A). MTS (Figure 2B, 2C) assay and Colony formation assay (Figure 2D, 2E) showed that knockdown Lucat1 inhibited cell proliferation in ACHN, 786-O and OS-RC-2 cells. Further assay of transwell showed that knockdown Lucat1 suppressed renal cancer cell migration and invasion (Figure 2F, 2G, 2H and 2I). Moreover, knockdown Lucat1 expression resulted in an accumulation of ACHN and 786-O cells in the G1 phase of the cell cycle (Figure 2J, 2K). In ccRCC cell lines OS-RC-2, we get the similar result (Supplementary Figure 1). We also found that up-regulation of Lucat1 can promote ccRCC proliferation *in vitro* (Suppementary Figure S1).

### Lucat1 inhibits p57 expression via directly binding with PRC2 complex in ccRCC cells

**Figure 3:**
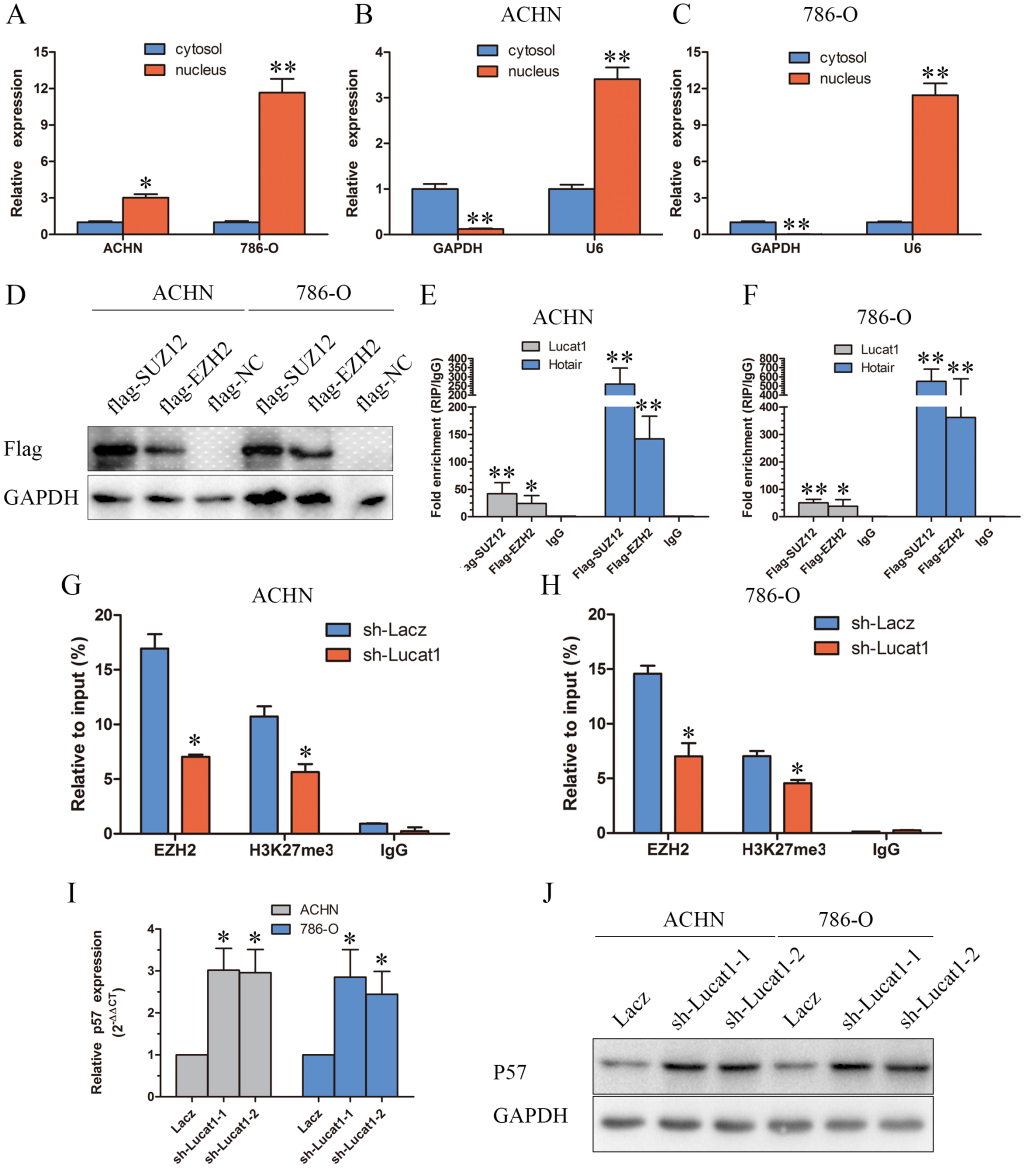
Lucat is associated with PRC2 in ccRCC **(A, B** and **C)** Lucat1 nuclear localization, as identified using qRT-PCR in fractionated ACHN and 786-O cells. After nuclear and cytosolic separation, RNA expression levels were measured by qRT-PCR. GAPDH was used as a cytosolic marker, and U6 was used as a nuclear marker. **(D)** ACHN and 768-O cells transfected with psi-Flag (control), or Flag-EZH2 and Flag-SUZ12 plasmids. **(E, F)** RIP experiments were performed with Flag antibody, and the coprecipitated RNA was subjected to qRT-PCR for Lucat1 and Hotair. The fold enrichment of Lucat1 and Hotair in RIPs is relative to its matching IgG control RIP. **(G, H)** ChIP-qPCR of H3K27me3 and EZH2 of the promoter region of the p57 locus after between ACHN and 786-O cell transfected with sh-Lacz or sh-Lucat1. Antibody enrichment was quantified relative to the amount of input DNA. Antibody directed against IgG was used as a negative control. **(I)** qRT-PCR detected the expression of p57 after transfection with sh-Lacz or sh-Lucat1. **(J)** Western blot assays detected the expression of p57 after transfection with sh-Lacz or sh-Lucat1.

Lucat1 is a long intergenic noncoding RNA, and it is estimated that up to 20% lincRNAs physically associate with PRC2 [12]. Thus, we hypothesized that Lucat1 might function in such a manner in ccRCC. As for PRC2 complex exerts its epigenetic functions in the nucleus, we first investigated the subcellular localization of Lucat1 in ccRCC and found that a considerable increase in Lucat1 expression in the nucleus versus the cytosol (Figure 3A, 3B, 3C), suggesting that Lucat1 may have a major regulatory function at the transcriptional level through PRC2. Furthermore, we performed RIP assay and the results showed that Lucat1 could directly bind with enhancer of zeste homolog 2 (EZH2, the catalytic subunit of the PRC2) and SUZ12, another member of the PRC2 complex in ACHN and 786-O cells (Figure 3D, 3E, 3F). We also found that another long non-coding RNA Hotair could bind with EZH2 and SUZ12 as previously reported. Previous studies showed that the expression of PRC2 target genes, such as the cell-cycle regulation genes CDKN1A (p21CIP1), CDKN1B (p27KIP1), CDKN1C (p57Kip2), CDKN2A (p16INK4a), CDKN2B (p15INK4b) were up-regulated at the mRNA and protein levels [14, 29]. We then graphed a correlation between Lucat1 and CDKN1A (p21CIP1), CDKN1B (p27KIP1), CDKN1C (p57Kip2), CDKN2A (p16INK4a), CDKN2B (p15INK4b) in TCGA data, and found only CDKN1C had a negative correlation with Lucat1 (Supplementray Figure 2). And then we performed a correlation between EZH2 and CDKN1A (p21CIP1), CDKN1B (p27KIP1), CDKN1C (p57Kip2), CDKN2A (p16INK4a), CDKN2B (p15INK4b) in TCGA data and obtained the same result (Supplementray Figure 2). So we mainly focused on Lucat1, EHZ2 and p57. To address whether Lucat1 is involved in transcriptional repression through the enrichment of PRC2 to p57 promoters, we conducted chromatin immunoprecipitation (ChIP) analysis by Lucat1 knockdown. ChIP assays demonstrated that knockdown of Lucat1 decreased the binding of EZH2 and H3K27me3 levels across the p57 promoters (Figure 3G, 3H). We next explored the role of Lucat1 on the expression of p57. The results disclosed that Lucat1 knockdown increased the expression of p57 at the mRNA and protein levels (Figure 3I, 3J).

### Silencing of p57 is partly involved in the oncogenic function of Lucat1

**Figure 4:**
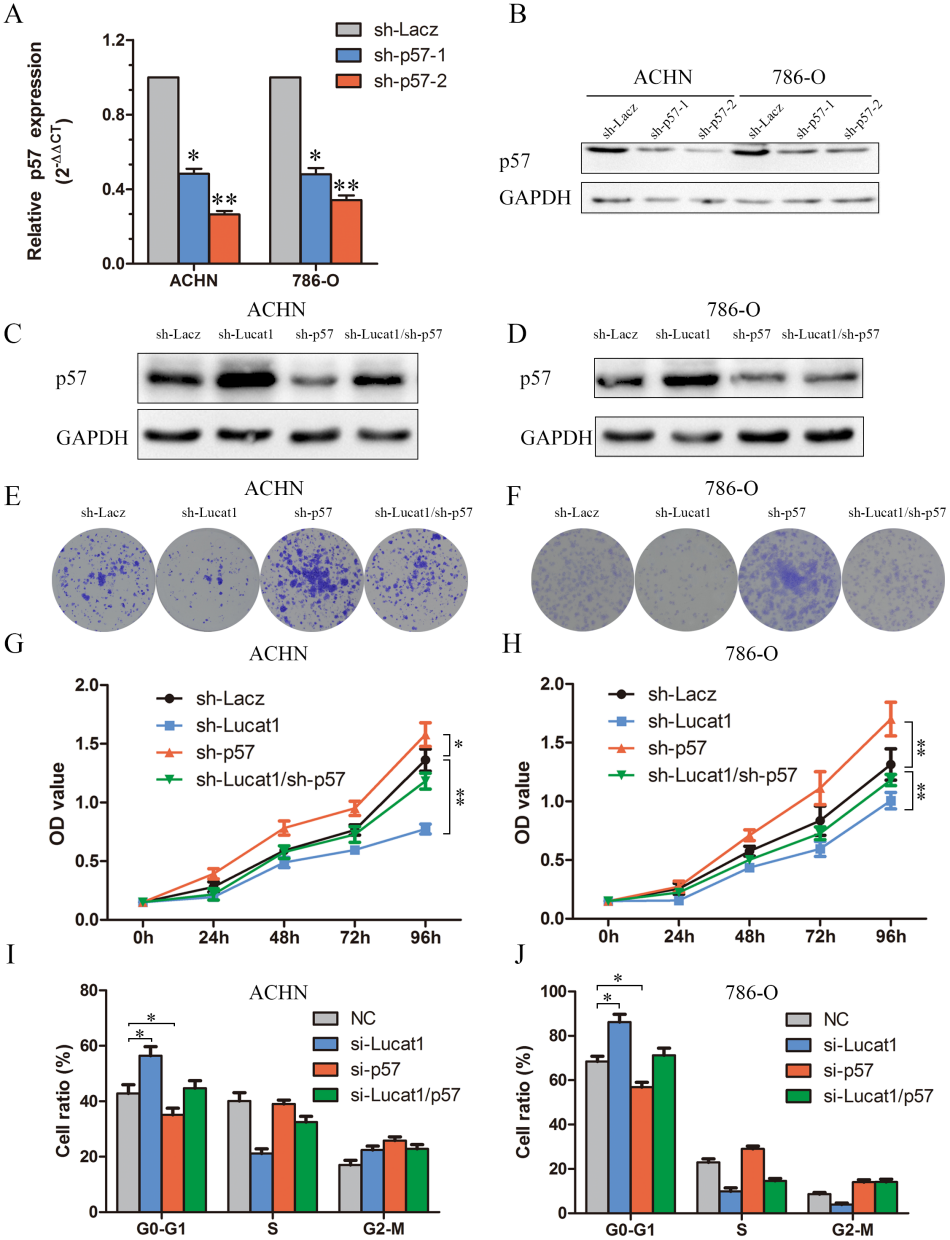
Silencing of p57 is partly involved in the oncogenic function of Lucat1 **(A, B)** The effciency of p57 silencing in short hairpin RNA-stably transduced renal cancer cell lines ACHN and 786-O by qRT-PCR and western blot. **(C, D)** Western blot assays detected the expression of p57 after transfection with sh-Lucat1 and/or sh-P57 in ACHN and 768-O. **(E, F)** Colony forming assays were performed to determine the cell viability in ACHN and 786-O cell lines. **(G, H)** MTS assays were performed to determine the cell viability in ACHN and 786-O cell lines. **(I, J)** Flow cytometric determination of proportion of indicated cells in distinct cell cycle phases.

We conducted rescue assays to identify whether p57 is mediated in the Lucat1 induced ccRCC cell growth. We stably inhibited p57 in two ccRCC cell lines ACHN and 786-O with lenti-viruses carrying shRNA for p57 and a control nonspecifc shRNA (LacZ) (Figure 4A, 4B). The effects of Lucat1 expression on endogenous p57 protein were monitored. It showed that sh-Lucat1 can promote the expression of p57, whereas sh-p57 can relieve the promotion of p57 by Lucat1 in renal cancer cell lines ACHN and 768-O (Figure 4C and 4D). Colony formation assay and MTS demonstrated that the knockdown of Lucat1 inhibited cell growth was partly reversed by sh-p57 treatment (Figure 4E, 4F, 4G, 4H and Supplementary Figure 3). Further study showed that knockdown of Lucat1 lead G0-1 cycle arrest was partly reversed by down-regulation of p57 (Figure 4I and 4J).

These findings indicated that Lucat1 exerts oncogenic effects in ccRCC cells partly through repressing p57 expression.

### Knockout of EZH2 is involved in the oncogenic function of Lucat1

**Figure 5:**
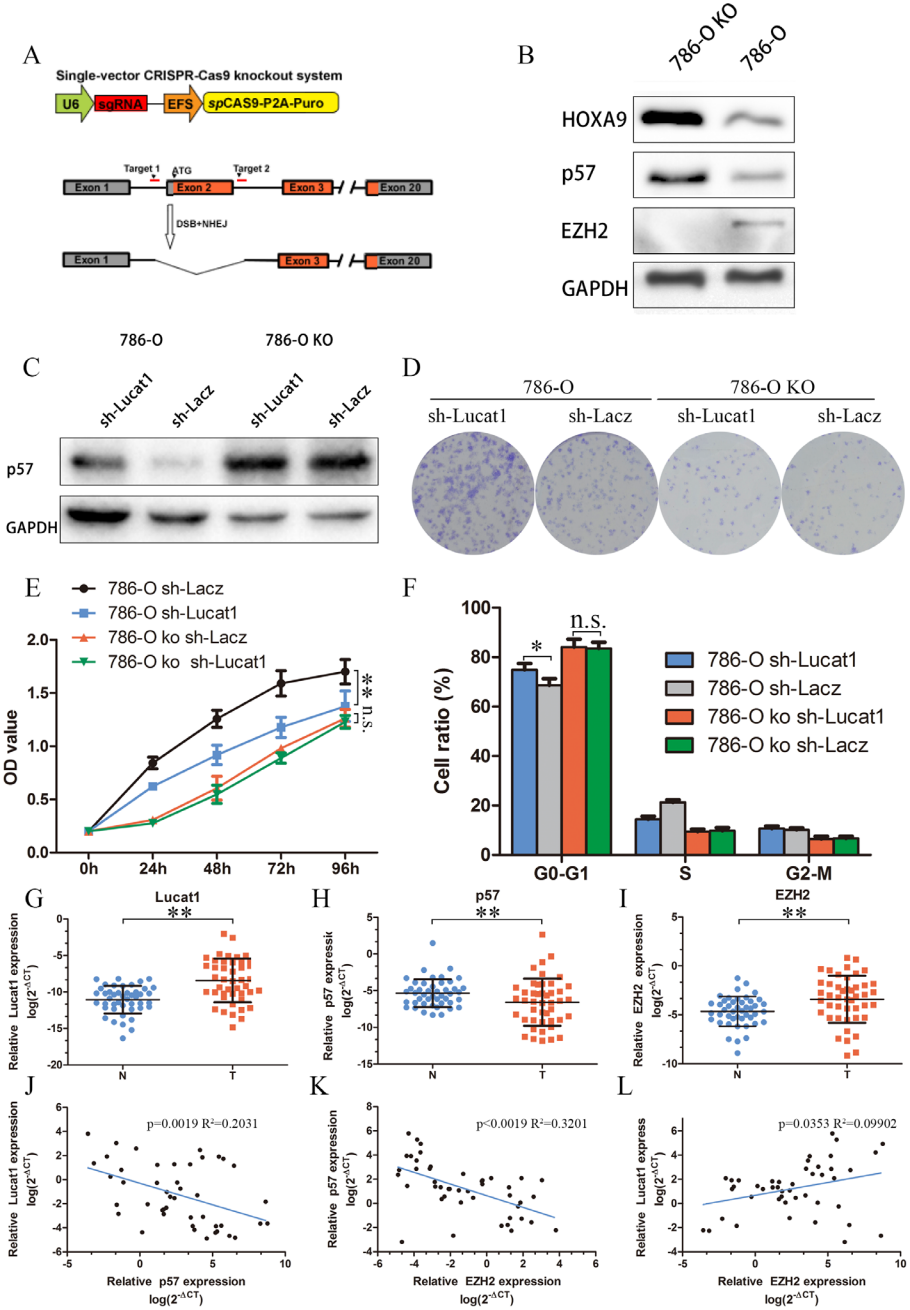
Lucat1 have its function through EZH2 **(A)** Schematic Model of knockout EZH2 with CRISPR/Cas9 system. **(B)** Western blot for EZH2, p57 and HOXA9 in 786-O EZH2 knockout cell lines and its control cell lines. **(C)** Western blot assays detected the expression of p57 after transfection with sh-Lucat1 and/or sh-P57 in 768-O and 786-O knockout cell lines. **(D, E, F)** Colony forming assays, MTS assays and flow cytometric were performed to determine the cell viability in 786-O and 786-O EZH2 knockout cell lines. **(G, H, I)** Relative expression of EZH2, P57 in 45 pairs of ccRCC tumor tissues and their corresponding adjacent non-cancerous tissues. **(J, K, L)** The correlation of Lucat1, EZH2 and P57 in renal cancer tissue.

CDKN1C (p57) is a direct target of EZH2 and is suppressed by epigenetic mechanisms in breast cancer, ovarian cancer, non-small-cell lung cancer etc. [30, 31]. But the mechanism in ccRCC remains unclear. We applied the efficient CRISPR/Cas genome editing system [32] targeting exon 2 of EZH2 to knock out this gene in 786-O cells [26]. Consistent with previous reports, EZH2-null clones exhibited a significant increase in p57 and HOXA9 (also a direct target of EZH2) level comparing to controls (Figure 5A, 5B). We then stably transfected sh-Lacz and sh-Lucat1 in 786-O EZH2 knockout cell lines and found that stably transfected sh-Lucat1 can’t clearly inhibit the expression of p57 (Figure 5C). Colony assay, MTS and cycle test also showed that sh-Lucat1 did not exist obvious effects on proliferation in 786-O EZH2 knockout cell lines (Figure 5D, 5E, 5F and Supplementary Figure 3). These findings indicated that Lucat1 exertion of oncogenic effects in ccRCC cells may be accomplished mainly through interaction with EZH2.

Next, we studied the clinic-pathological relevance of the Lucat1, its relative molecule p57, and EZH2 expression in 45 matched normal and ccRCC clinical tissue samples. We found significantly elevated expression of EZH2, Lucat1 and a lower expression of p57 in the tumor samples (Figure 5G, 5H and 5I). Bivariate correlation analysis showed that expression of Lucat1 and EZH2 were significantly correlated with p57 transcript level of ccRCC tissues (Figure 5J, 5K), but there was low correlation between Lucat1 and EZH2 (Figure 5L).

### Knockdown of Lucat1 represses tumor growth *in vivo*

**Figure 6:**
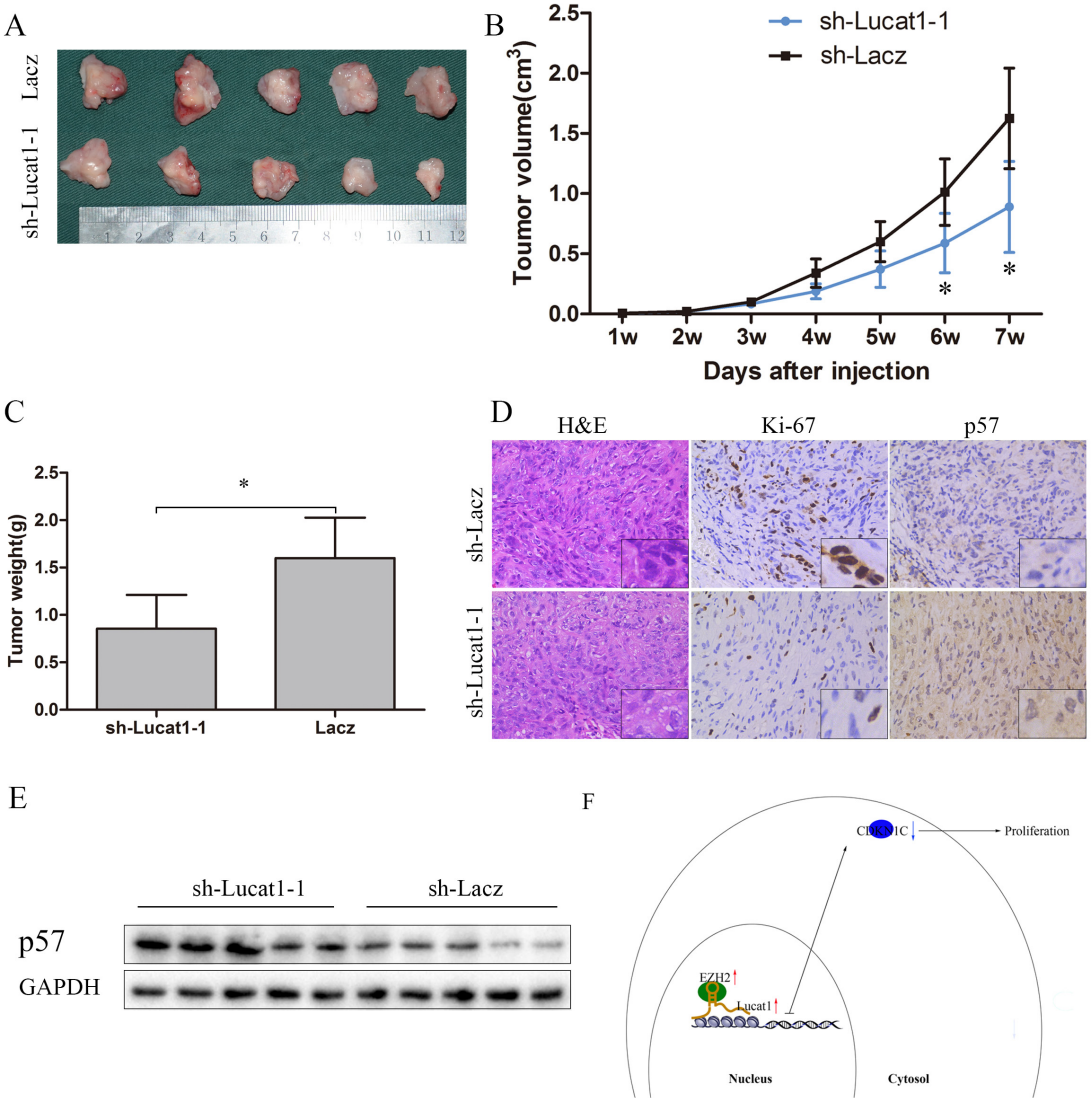
Lucat1 induced proliferation *in vivo* **(A)** Photographs of tumors excised 7 weeks after inoculation of stably transfected cells ACHN into the armpit of nude mice. **(B)** Mean tumor volume measured by caliper on the indicated days. **(C)** Tumor weight of each nude mouse at the end of 7 weeks. **(D)** IHC staining for H&E, ki-67 and p57 in slices of sectioned implanted tumors formed by indicated cells. Original magnifcation was ×200. **(E)** Western blotting confirmed protein expression of p57 in indicated tumors. **(F)** Schematic Model of Long noncoding RNA Lucat1 acts as an oncogene in clear-cell renal cell carcinoma by binding to polycomb PRC2 complex and repressing p57.

To further determine whether Lucat1 affects tumorigenesis *in vivo*, sh-Lacz/sh-Lucat1 transfected ACHN cells were inoculated into nude mice. Up to 49 days, there was a dramatic decrease in tumor volume and weight in the sh-Lucat1 group compared with sh-LacZ group (Figure 6A, 6B and 6C). Moreover, we also found that the tumors developed from sh-Lacz cells showed stronger Ki-67 expression than tumors formed from sh-Lucat1 and that tumors that developed from sh-Lucat1 cells showed a stronger p57 expression than tumors formed in the control, as detected by IHC analysis (Figure 6D). In the fresh subcutaneous tumor, we detected the expression of p57 by Western Blot. There was higher expression of p57 in sh-Lucat1 group than the sh-Lacz group (Figure 6E).

These results suggest that the level of Lucat1 expression is significantly associated with the proliferation capacity of ccRCC *in vivo* and may through decrease the expression of p57 (Figure 6F).

## DISCUSSION

Recently, many studies have shown that lncRNAs are frequently in dysregulation in various tumors and have multiple functions in a wide range of biological processes, such as proliferation, apoptosis, cell cycle arrest or cell migration and invasion [5, 33]. Given that Lucat1is induced by cigarette smoke and elevated in lung cancer cell lines and may contribute to cisplatin resistance in high grade serous ovarian cancer, we speculated that Lucat1 might involve in the ccRCC progression. With the aid of high-throughput techniques, especially the TCGA data, we identified and characterized Lucat1 as an oncogenic lncRNA in ccRCC. The results revealed that Lucat1 was upregulated in ccRCC tumorous tissues and markedly correlated with poor prognosis shorter overall survival and progression free survival. What’s more, silencing Lucat1 also impaired cell proliferation, migration and invasion, and cell cycle arrest *in vitro*, and inhibited tumorigenesis of ccRCC cells *in vivo*.

Many lncRNAs affect this aspect of cell transformation, as they interface with the epigenetic machinery influencing gene expression. Several can associate with the polycomb repressive complex 2 (PRC2), a chromatin repressor complex that catalyzes H3K27 trimethylation and which is tightly linked to the aberrant proliferation of cancer cells. Some lncRNAs affect gene expression through post-transcriptional processes, including splicing turnover, exportation or translation of mRNAs, as well as the stability and post-translational modification of proteins [33]. P57 is an inhibitor for cyclin-dependent kinase, and is deemed as a candidate for a tumor-suppressive gene that has been embroiled in numerous of cancers [31, 34, 35]. Bishoy A *et.al* examined the expression of p16, p21, p27, p53 and p57 in a large cohort of patients in ccRCC treated with extirpative therapy, and found that p57 was the most common altered marker, altered in 60% of 452 patients [35]. The TCGA data also showed that p57 exhibited a lower expression in tumor than in adjacent normal tissue in urothelial bladder cancer, breast cancer, colon and rectal adenocarcinoma, chromophobe renal cell carcinoma, ccRCC, lung adenocarcinoma etc. In addition, we also found that knockdown of Lucat1 increased the expression of p57 in ccRCC cells. Furthermore, we found that knockdown of Lucat1 inhibited cell proliferation, migration and invasion, and cell cycle arrest, while knockdown of p57 reversed the negative role of cell proliferation, and cell cycle arrest in ACHN and 786-O cell lines, which indicated p57 was a novel Lucat1 target, and Lucat1 could function as oncogene through suppressing p57 expression in ccRCC cells. Many lncRNAs affect the expression of p57 through recruiting EZH2 [14, 23, 36-38].

We applied the efficient CRISPR/Cas genome editing system targeting exon 2 of EZH2 to knock out this gene in 786-O cells [26] and found a higher expression of p57 in the EZH2-knockout cell lines. Moreover, we also found that in EZH2-knockout cell lines, Lucat1 cannot affect the expression of p57 and the proliferation of ccRCC, which further verified that Lucat1 decrease the expression of p57 through EZH2.

We have tried to detect the genes that can regulate the expression of Lucat1 in ccRCC, and found that the PTEN mutant group exhibited a higher expression of Lucat1 than the PTEN wide-type group from TCGA (supplementary Figure 4). Further analysis showed that Lucat1 has the strongest positive correlation with miR-21 (Supplemental Figure 4, Supplementary Table 2), a clearly reported target for PTEN. However, down-regulation of PTEN or miR-21 exhibited no obvious effects on the expression of Lucat1 (data not shown). Further investigation for genes relative to the regulation of the expression of Lucat1 is needed. Thus, it is reasonable for us to conclude that Lucat1 promotes ccRCC cell proliferation through recruitment of the Polycomb PRC2 complex and inhibition of p57.

In summary, our results provide strong evidence that Lucat1 is up-regulated in ccRCC tumor tissues and cell lines, and is associated with poor prognosis in ccRCC patients. Lucat1 acts as an oncogene in clear-cell renal cell carcinoma by binding to polycomb PRC2 complex and suppressing p57. This lncRNA may serve as a target for novel therapies in ccRCC.

## MATERIALS AND METHODS

### Clinical sample preparation

A total of 45 paired clear cell renal cell carcinoma and corresponding noncancerous tissues were obtained sequentially from patients undergoing radical nephrectomy during the period of 2010–2016 in Union hospital. Corresponding noncancerous tissues were acquired at least 5cm away from the tumor site. The study protocol was approved by the ethics committee of Huazhong University of Science and Technology and Union Hospital, and a written informed consent form was obtained from all participants involved in this study.

### Cell culture

ACHN, 786-O and HK-2 cells were maintained in Dulbecco’s modified Eagle’s medium, supplemented with 10% fetal bovine serum and 2 mmol/L l-glutamine in a humidified atmosphere of 5% CO2 maintained at 37 °C. OS-RC-2 and CaKi-1 cells were cultured in RPMI-1640 supplemented with 10% fetal bovine serum and 2 mmol/L l-glutamine [24].

### TCGA analysis

The RNA-seq data of 448 tumours and 67 matched normal samples were downloaded from The Cancer Genome Atlas (TCGA) Data Portal (http://ibl.mdanderson.org/tanric/_design/basic/query.html) [25].

### Oligonucleotide, lentivirus, plasmid and shRNA

Oligos corresponding to the target sequences were annealed and cloned into the AgeI and EcoRI sites of the plko.l plasmid (Addgene).

### Quantitative real-time PCR (RT-qPCR)

All RNA from tissues and cells were extracted with the TRIzol reagent (Invitrogen, Carlsbad, CA) according to the manufacturer’s protocol with modifcation. cDNAs were synthesized using Rever Ace qPCR RT Kit (TOYOBO). Real-time PCR was performed using SYBR Green Realtime PCR Master Mix (Roche) and the ABI ViiA7 qPCR System (Applied Biosystems). Chromatin immunoprecipitation (ChIP) was performed to investigate whether EZH2 and H3K27 binding to p57 promoter. ChIP assays were performed as described previously [26]. RNA binding protein immunoprecipitation (RIP) experiments were performed using Magna RIP Kit (Millipore, Catalog No.17-701) according to the manufacturer’s instructions and a previously published RIP-Chip protocol [27]. RIP assays were carried out as described previously [26]. All primers were in Supplementary Table 1.

### Colony formation, cell proliferation assay, cell cycle analysis, cell migration and invasion

Colony formation was measured nearly two weeks after seeding 1000 cells per well in 6-well plates. Cell proliferation was estimated using the MTS according to manufacturer instructions. Fluorescence-activated cell-sorting (FACS) (BD, USA) analysis was done using propidiumiodide (PI) stains for cell-cycle analysis according to the manufacturer’s protocol with three replications [28]. Migration and invasion assays were performed using uncoated and Matrigel™ coated Transwell^®^ inserts according to manufacturer instructions. 10^5^ and 1.5^*^10^5^ cells for ACHN migration and invasion. 3^*^10^4^ and 5^*^10^4^ cells for 786-O migration and invasion. All experiments were triplicated [26].

### Xenograft subcutaneously

Tumorgenesis in nude mice was determined as described previously [5]. Five mice each were injected subcutaneously with prepared cells at a single site. Tumor onset was measured with calipers at the site of injection weekly at different times on the same day. All experiments were approved by the Animal Care and Use Committee of Tongji Medical College of Huazhong University of Science and Technology.

### Immunohistochemical staining

As previously described [28].

### Statistical analysis

All statistical analyses were carried out using SPSS 18.0 statistical software. Continuous data were compared using Student’s 2-tailed t-test. Data are represented as mean ± SEM. In all cases, p < 0.05 was considered statistically significant. ^*^p < 0.05;^**^p < 0.01.

## SUPPLEMENTARY MATERIALS.FIGURES AND TABLES



## Abbreviations

ccRCC: clear cell renal cell carcinoma
Lucat1: lung cancer associated transcript 1
lncRNA: long non-coding RNA
PRC2: polycomb repressive complex 2
