# Supplementary material for: Long non-coding RNA Lucat1 is a poor prognostic factor and demonstrates malignant biological behavior in clear cell renal cell carcinoma
Source: Oncotarget. 2017 Sep 23;8(69):113622–34. doi: 10.18632/oncotarget.21185 (PMC5768351; doi:10.18632/oncotarget.21185)
Supplement: Supplementary file 1 [file oncotarget-08-113622-s001.pdf]

Long non-coding RNA Lucat1 is a poor prognostic factor and demonstrates malignant biological behavior in clear cell renal cell carcinoma

SUPPLEMENTARY MATERIALS

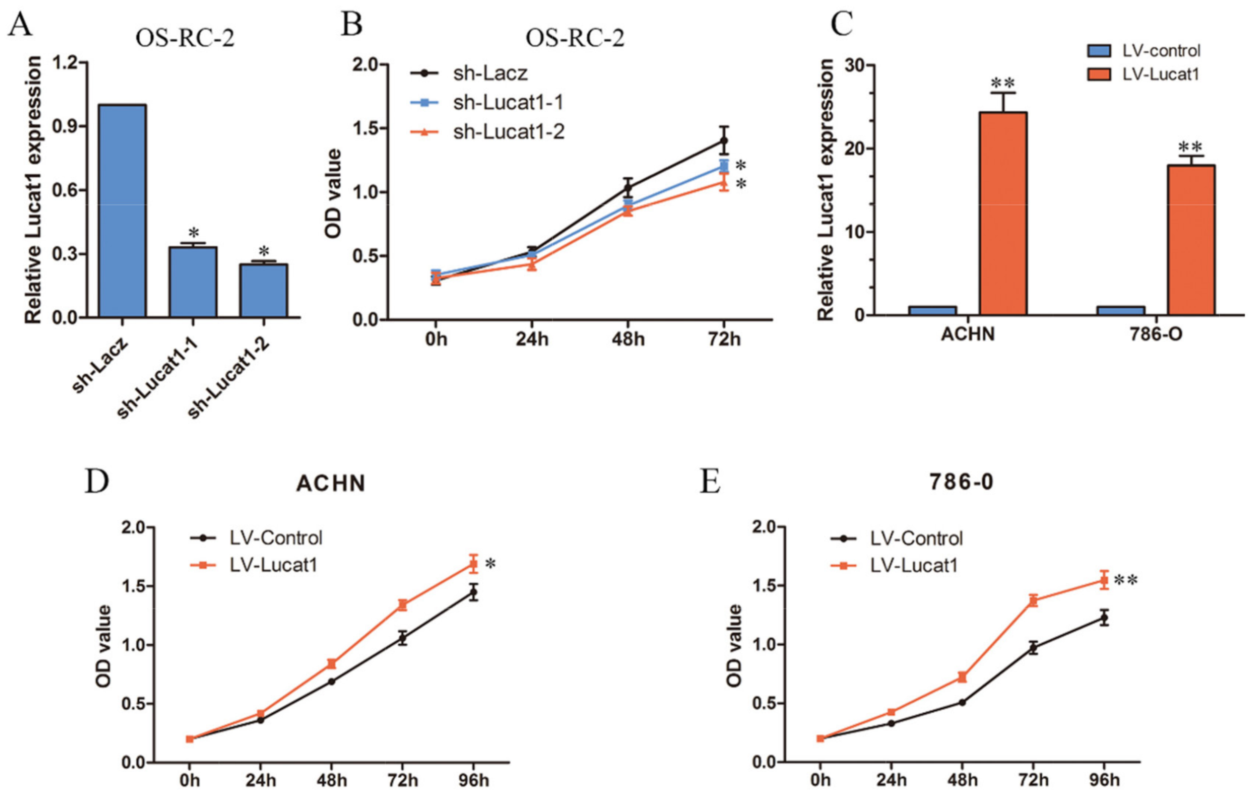

Supplymentary Figure 1: Lucat1 inhibited cell proliferation *in vitro*. (A) The efficiency of Lucat1 silencing in short hairpin RNA-stably transduced renal cancer cell lines OS-RC-2. Relative gene expression was determined using the comparative delta-delta CT method (2-ΔΔCt). **(B)** MTS assays revealed cell growth curves of indicated cells. **(C)** The efficiency of up-regulation Lucat1 expression in renal cancer cell lines ACHN and 786-O. **(D, E)** MTS assays revealed cell growth curves of indicated cells.

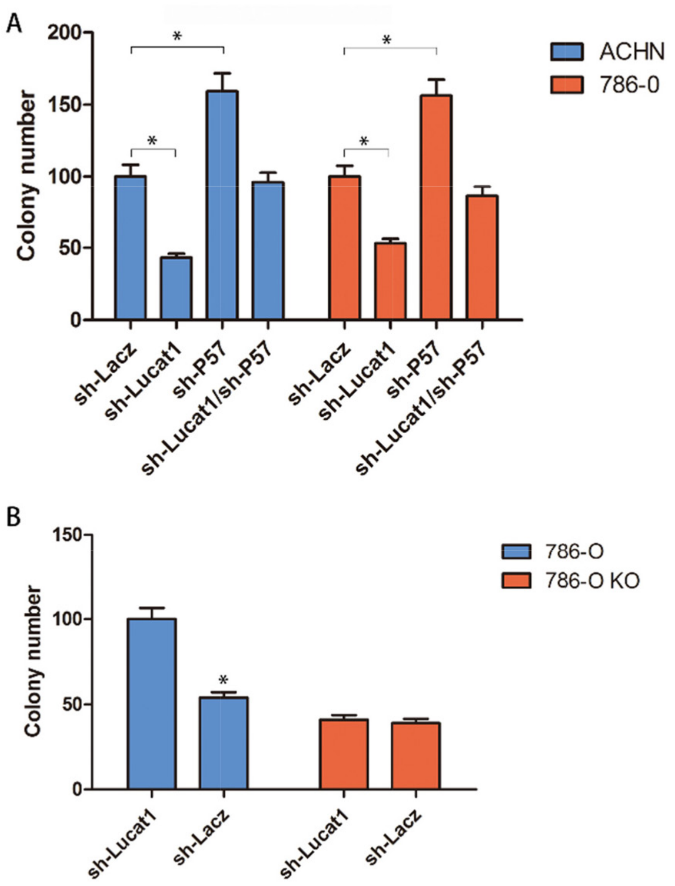

Supplymentary Figure 2: The relative count of Representative micrographs of crystal violet-stained cell colonies. (A): The relative count colonies in Figure 4E and 4F; **(B)**: The relative count colonies in Figure 5D.

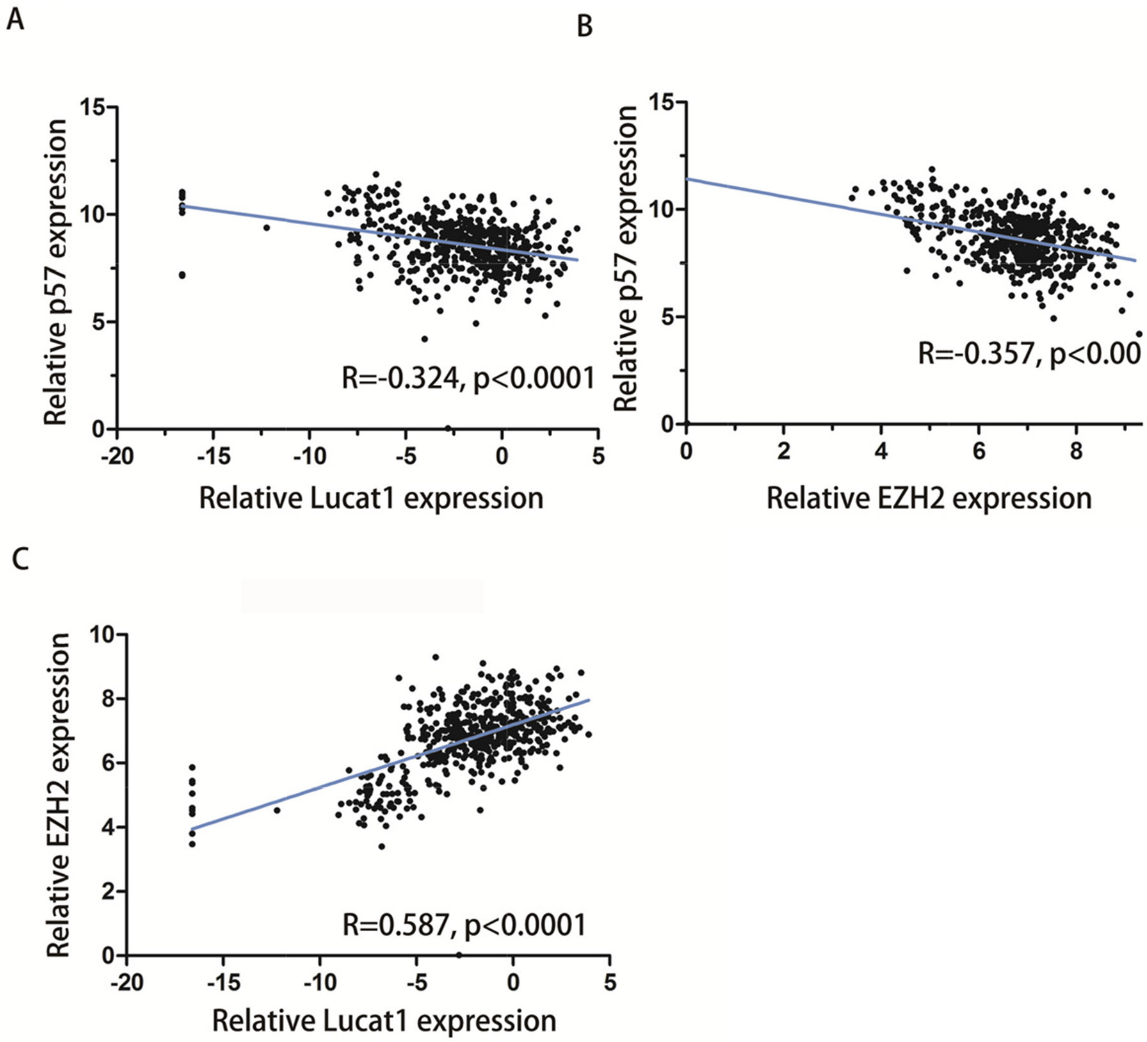

Supplymentary Figure 3: The correlation between EZH2, Lucat1 and p57. **(A)** The correlation between p57 and Lucat1. **(B)** The correlation between p57 and EZH2. **(C)** The correlation between Lucat1 and EZH2.

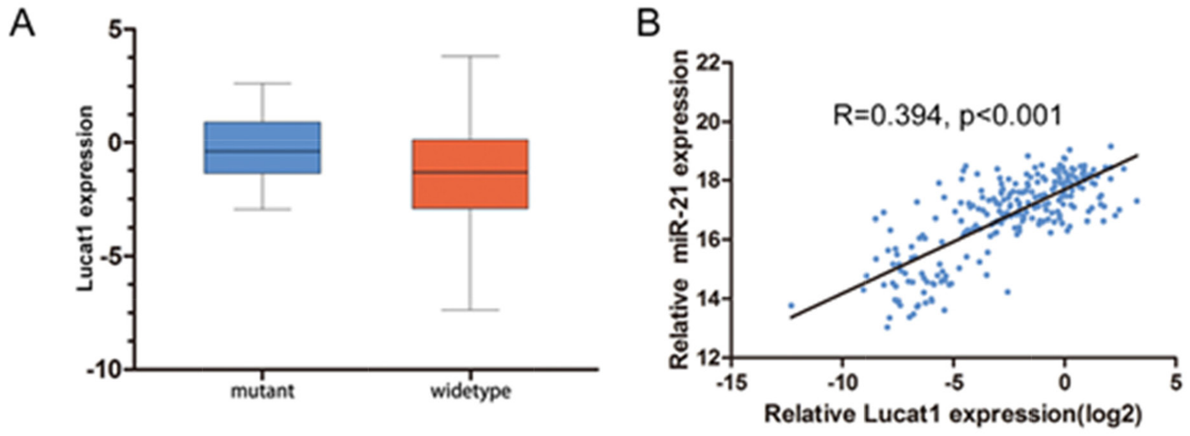

Supplymentary Figure 4: The correlation between Lucat1, PTEN and miR-21. **(A)** The status of PTEN and the expression of Lucat1. **(B)** The correlation between Lucat1 and miR-21.

Supplymentary Table 1: The primers used in the study

GENE	Forward primer	Reverse primer
**human qRT-PCR**		
Lucat1	ACCAGCTGTCCCTCAGTGTTCT	AGGCCTTTATCCTCGGGTTGCCT
Hotair	CCAGAGTTACAGACGGCGG	CTACCAGGTCGGTACTGGCTT
p57	CTAGCCAGCAGGCATCGAG	GTGGTGGACTCTTCTGCGTC
EZH2	TTCTGTGCCATTGCTAGGTTA	CTTTTTCCTTGGAGGAGTATC
GAPDH	CCTTCATTGACCTCAACTACA	GCTCCTGGAAGATGGTGAT
U6	CTCGCTTCGGCAGCACA	AACGCTTCACGAATTTGCGT
**ChIP primers**		
**p57**	TCCATCTACTGGGCAGGGTG	ACAGGGGTCAGCTCCACTCT
**gRNA primer**		
**EZH2**	CACCGACACGCTTCCGCCAACAAAC	AAACGTTTGTTGGCGGAAGCGTGTC
**shRNA**		
**sh-Lucat1-1**	GAGATTATTTAGTGACTGAAATGTTTTTTGC<br>TCGAGTACTAGGATCCATTAGGC	TTGAATTATTTAGTGACTGAAATGAAACAAG<br>GCTTTTCTCCAAGGGATATTTATA
**sh-Lucat1-2**	GAGAAGTAGAACACTGAGGGACAGCTTTTTTGC<br>TCGAGTACTAGGATCCATTAGGC	TTGAAAGTAGAACACTGAGGGACAGCAAACAAG<br>GCTTTTCTCCAAGGGATATTTATA
**sh-p57-1**	GAGAAAGTCGTAATCCCAGCGGTTCTTTTTTGC<br>TCGAGTACTAGGATCCATTAGGC	TTGAAAAGTCGTAATCCCAGCGGTTCAAACAAG<br>GCTTTTCTCCAAGGGATATTTATA
**sh-p57-2**	GAGAAATAACCGAGCTAGTGCGTGGTTTTTTGC<br>TCGAGTACTAGGATCCATTAGGC	TTGAAAATAACCGAGCTAGTGCGTGGAAACAAG<br>GCTTTTCTCCAAGGGATATTTATA

Supplymentary Table 2: The correlation between Lucat1 and miRNAs in TCGA (top 20)

Lucat1	R	P value
hsa-mir-21	0.394191096	<0.001
hsa-mir-122	0.355840005	<0.001
hsa-mir-3941	0.352768117	<0.001
hsa-mir-590	0.348043542	<0.001
hsa-mir-25	0.347494421	<0.001
hsa-mir-550a-1	0.339270695	<0.001
hsa-mir-28	0.338490398	<0.001
hsa-mir-18a	0.324258078	<0.001
hsa-mir-19a	0.320599997	<0.001
hsa-mir-629	0.314114586	<0.001
hsa-mir-3613	0.31319034	<0.001
hsa-mir-224	0.313154191	<0.001
hsa-mir-210	0.313143778	<0.001
hsa-mir-1228	0.313096343	<0.001
hsa-mir-155	0.306745737	<0.001
hsa-mir-2355	0.301227743	<0.001
hsa-mir-106b	0.29585982	<0.001
hsa-let-7i	0.294165159	<0.001
hsa-mir-15a	0.291259444	<0.001
hsa-mir-550a-2	0.288657011	<0.001
